# Genetic basis for hyper production of hyaluronic acid in natural and engineered microorganisms

**DOI:** 10.1186/s12934-016-0517-4

**Published:** 2016-07-01

**Authors:** Juliana Davies de Oliveira, Lucas Silva Carvalho, Antônio Milton Vieira Gomes, Lúcio Rezende Queiroz, Beatriz Simas Magalhães, Nádia Skorupa Parachin

**Affiliations:** Pós-Graduação em Ciências Genômicas e Biotecnologia, Universidade Católica de Brasília, Brasília, DF CEP 70.790-160 Brazil; Integra Bioprocessos e Análises, Campus Universitário Darcy Ribeiro, Edifício CDT, Sala AT-36/37, Brasília, DF CEP 70.904-970 Brazil; Grupo de Engenharia Metabólica Aplicada a Bioprocessos, Instituto de Ciências Biológicas, Universidade de Brasília, Brasília, DF CEP 70.790-900 Brazil

**Keywords:** Hyaluronic acid, Hyaluron synthase, *Streptococcus zooepidemicus*, *Bacillus subtillis*, *Pichia pastoris*, *Pasteurella multocida*, *Escherichia coli*

## Abstract

Hyaluronic acid, or HA, is a rigid and linear biopolymer belonging to the class of the glycosaminoglycans, and composed of repeating units of the monosaccharides glucuronic acid and *N*-acetylglucosamine. HA has multiple important functions in the human body, due to its properties such as bio-compatibility, lubricity and hydrophilicity, it is widely applied in the biomedical, food, health and cosmetic fields. The growing interest in this molecule has motivated the discovery of new ways of obtaining it. Traditionally, HA has been extracted from rooster comb-like animal tissues. However, due to legislation laws HA is now being produced by bacterial fermentation using *Streptococcus zooepidemicus*, a natural producer of HA, despite it being a pathogenic microorganism. With the expansion of new genetic engineering technologies, the use of organisms that are non-natural producers of HA has also made it possible to obtain such a polymer. Most of the published reviews have focused on HA formulation and its effects on different body tissues, whereas very few of them describe the microbial basis of HA production. Therefore, for the first time this review has compiled the molecular and genetic bases for natural HA production in microorganisms together with the main strategies employed for heterologous production of HA.

## Background

**Fig. 1:**
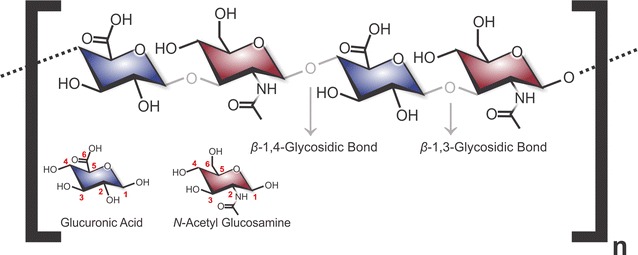
Molecular structure of repeating disaccharide units composed of *N*-acetylglucosamine and glucuronic acid, which leads to the formation hyaluronic acid (HA). Each unit is also depicted, with the position of each carbon atom indicated by a *number*

Hyaluronic acid, also called hyaluronan or hyaluronate (HA), is considered an important glycosaminoglycans due to its varied physiological functions. This polymer is composed of disaccharide repetitions of glucuronic acid (UDP-GlcUA) and *N*-acetylglucosamine (UDP-GlcNAc), linked by β1 → 3 and β1 → 4 glycosidic bonds (Fig. 1) [1, 2]. In contrast to other glycosaminoglucans, HA is the only non-sulphated polymer, which allows the molecule to be rigid and straight [1]. It is produced in the plasma membrane of all mammalian cells, amphibians and bacteria [3] by integral membrane enzymes called hyaluronic acid synthases (HAS), which have several isoforms according to its producing organism.

In humans, HA is present in all organs and especially abundant in connective tissue [4]. HA reaches higher concentrations in the cartilage tissue in the vitreous humor [5], the synovial fluid of the joints [6] and in the umbilical cord [7] and is responsible for the maintenance of tissue homeostasis [8]. This polymer is directly involved in processes such as embryogenesis [9], inflammation [10], metastasis or tumor progression phenomenon [11], angiogenesis [12] and the healing process [13]. Another common feature of HA in eukaryotic organisms is that it confers a smooth aspect to the skin. It has been reported that with aging, HA production decreases, which results in the dehydration and loss of elasticity of the skin, contributing to the appearance of wrinkles [14].

**Fig. 2:**
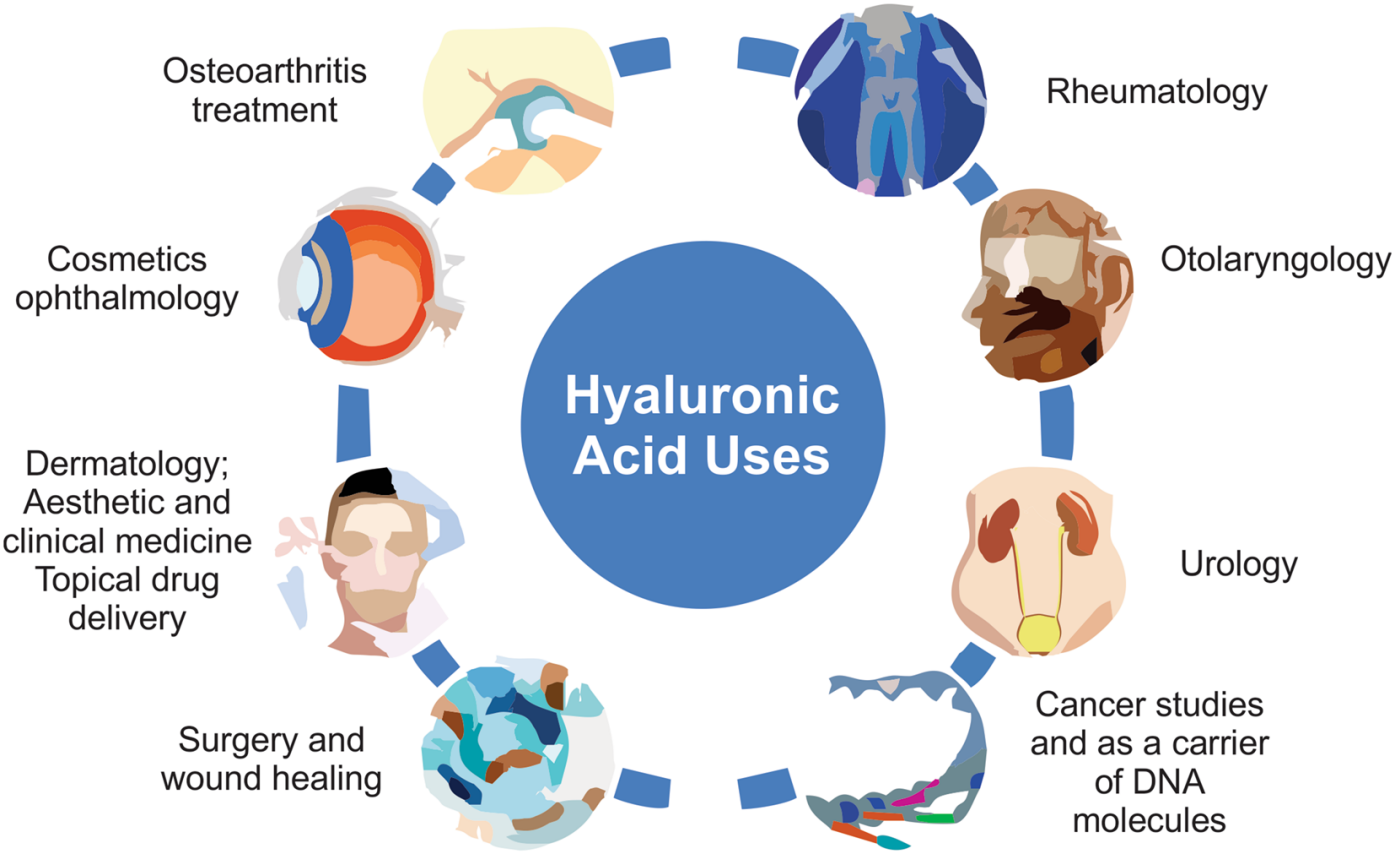
Biomedical applications of hyaluronic acid

The main characteristics of HA that makes it a very attractive molecule are: (1) high hygroscopicity; (2) viscoelastic nature; (3) high biocompatibility; (4) non-immunogenicity and (5) it does not generate toxic products when degraded. The use of HA has seen results in ophthalmic cosmetics [15], in surgery [16], as a drug delivery system [17], in rheumatology [18], in otolaryngology [19] and in urology [20]. The many applications of hyaluronic acid are illustrated in Fig. 2. However, of all the previously mentioned applications, the use of HA is more frequent in the field of dermatology as dermal filler for the treatment of wrinkles. Lastly, in tissue engineering, HA is used as a mechanically and physically appropriate support for tissue that can be implanted in organisms without causing allergic reactions or immune responses [21].

Together with the increased number of applications of hyaluronic acid, the market share tends to grow over the years. Currently, this polymer is valued at USD 1000–5000/kg depending on its purity and size [22]. According to a search conducted in 2014 by the intelligence company firm “Transparency Market Research”, the marketing value of the HA in 2012 was USD 5.32 billion and should reach USD 9.85 billion by 2019. Initially, its commercialization was done exclusively by extraction from animal tissues in the early 1940s [23]. Nevertheless, the disadvantages of this methodology include the loss of HA by degradation caused by the activity of the endogenous hyaluronidase enzyme, harsh extraction conditions and high purification costs, since animal-derived HA could contain contaminants such as viruses [23]. Therefore, alternative routes for HA production have been developed. To date, commercial hyaluronic acid is mainly obtained by the market through microbial fermentation. The use of HA from microorganisms is feasible since it is non-immunogenic and therefore biocompatible due to its highly-conserved structure among different species [24].

**Fig. 3:**
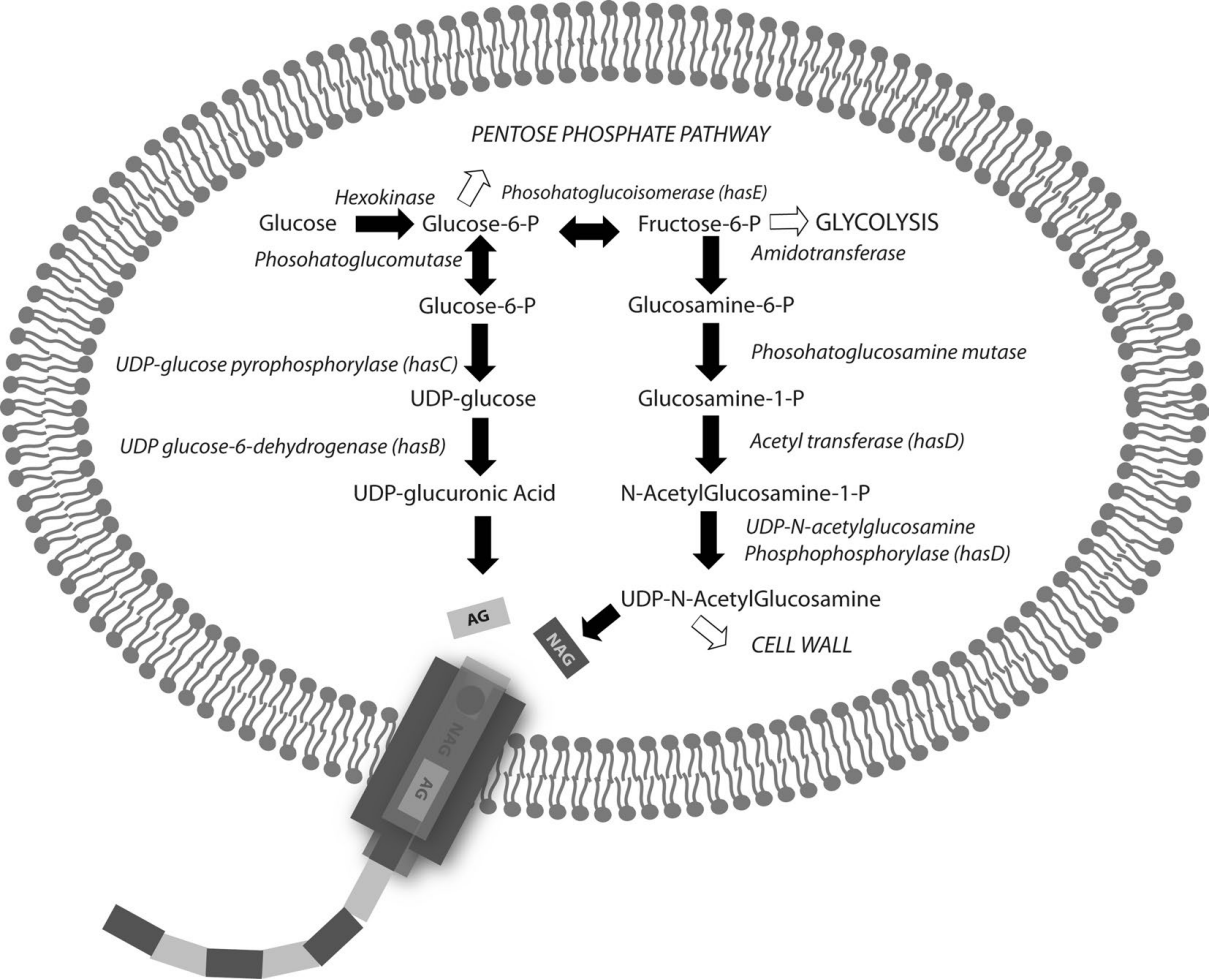
Biosynthetic pathway for hyaluronic acid in Streptococci. Some intermediates are also required for cell wall synthesis. Important genes are: *hasB* (coding for UDP-glucose 6-dehygrogenase); *hasC* (coding for glucose-1-P uridyltransferase); *hasA* (HA synthase)

In the early 80s, HA started to be produced using the bacteria *Streptococci* as a host cell. However, the genus *Streptococci* is known to possess several human pathogens, thus, the HA purification costs using this bacteria genus are elevated. Therefore, other microorganisms, natural producers or genetically engineered ones, have been considered for HA production. Ideally, a perfect microorganism for HA production should have GRAS status **(**generally regarded as safe**)**, not secrete any toxins and be able to produce the biopolymer continuously so it can reach at least 1 megadalton (MDa). The molecular weight (MW) and the purity of HA are indicative of its quality: polymers that have a greater MW (>0.5 MDa) have greater market value. From the microbial point of view, producing such a polymer is also a challenge because of its high metabolic energy cost. For instance, in order to produce a dimer of HA, three ATP molecules, two UTP molecules, two NAD^+^ molecules, one molecule of Acetyl-CoA and one molecule of glutamine (counting the energy expended towards glycolysis) are necessary for the synthesis of the two precursors of HA (Fig. 3).

The increased interest in HA production is reflected by the increased number of publications and patents filed over the last years. A search performed in databases has shown that 2220 articles and patents were published between 2005 and 2010. This number increased about 2.5 times in the period from 2011 to 2015. Considering the constant and increased interest in HA qualities, this review is aimed at summarizing the basis of HA production in naturally producing organisms and in engineered strains for hyper production. Most published reviews have focused on HA formulation and its effects in different body tissues, whereas very few of them describe the microbial basis of HA production [25–28]. Therefore, this review has compiled the genetic and molecular bases used by the microorganisms that are currently known to produce hyaluronic acid together with the main strategies employed for heterologous production of HA.

## Main text

### Basis of HA synthesis: HA-synthases

The synthesis of a HA chain in all natural producers initially emerge from the glycolytic pathway. The two precursors, UDP-GlcUA and UDP-GlcNAc are synthesized, respectively, from deviations of the molecules glucose-6-phosphate and fructose 6-phosphate from glycolysis (Fig. 3), in a process that occurs entirely on the inner side the plasma membrane in the cytoplasm. Furthermore, from the deviation of the glycolytic pathway to the production HA there is also the production of molecules used by cells as wall polysaccharide synthesis, synthesis of teichoic acids and peptidoglycan (Fig. 3) which corresponds to 20 % of dry weight of a regular cell [25]. In other words, the biomass formation competes with precursors to the synthesis of hyaluronic acid [29]. Once available in the cell, UDP-GlcUA and UDP-GlcNAc are used as substrates by hyaluronan synthase enzyme (HAS), an enzyme that have transmembrane domains and catalyze the union of the two sugar precursors intracellularly to finally release the chain in the extracellular matrix (Fig. 3) [30]. This process is advantageous to the cell since it allows the synthesis of unrestricted size chains, and prevent the waste energy cell for a possible subsequent transport chains out of the cell [31].

Although the elongation of hyaluronic acid chain through the plasma membrane is simple, HAS enzyme is an multidomain enzyme with six differente binding sites: (1) a UDP-GlcNAc binding site and (2) a UDP-GlcUA binding site, both for the capture of their precursors, (3) a domain GlcNAc β (1–4) transferase and (4) a domain UDP-GlcUA β (1–3) transferase which bind the precursors to each other and (5) an HA acceptor site which receives the newly formed chain and (6) excretes the molecule out of the cell [31].

A recent review detailed the main metabolic controlling factors of hyaluronan synthases [32]. HA is a glycosaminoglycan (GAG) synthesized outside the golgi, unlike other GAGs, and therefore dependent of the pool of precursors contained in the cytoplasm. Thus, the hyaluronic acid production is affected when both sugar nucleotide precursors are in low concentrations. On the other hand, an increase in the amount of precursors does not significantly affect the production of GAGs in the golgi, but affects the production of HA [32]. The majority of GAGs (which are sulfated) are synthesized in the golgi while the HA is synthesized at the plasma membrane, however, the affinity of the sugar nucleotides receptors located in the membrane of the golgi is very high. This feature makes the amount of sugars into the golgi is high all the time, unlike the amount of the cytoplasm [33, 34].

The UDP-GlcNAc is also an cell wall precursor and used by the cell in various other functions, coming to have a molar concentration equivalent to the molar ATP concentration [35, 36]. Thus, it is not unusual to observe that the amount of the UDP-GlcNAc inside the cell drastically affects the production of HA, even more than UDP-GlcUA. Previous studies have suggested that high concentrations of ATP and/or low concentrations of UDP-GlcNAc force the HAS enzyme to cleave the chain and release the polymer into the extracellular matrix [37]. Moreover, the proper balance in the synthesis of UDP-GlcNAc/UDP-GlcUA and the balance of the glycolytic rate and HA synthesis rate are also important factors for obtaining high molecular weight HA [38].

Not only the enzymes that produce directly both precursors affect the HA production, but also other enzymes, like UDP-glucose pyrophosphorylase, which converts glucose-1-phosphate to UDP-glucose is critical for the HA synthesis. The UDP-glucose is also a metabolite used in the reversible synthesis of glycogen, which can affect the energetic state of the cell to produce HA [39].

In metabolic terms, the demand for ATP, UTP and acetyl-CoA (Fig. 3) by the cell require an energy expenditure that is not always available and drastically affects the production of HA. Furthermore, the synthesis of 1 mol of UDP-GlcUA produces two moles of NADH, forcing the cell to find ways to recycle NAD^+^. In other words, a high rate NADH: NAD^+^ also inhibits HA production.

HASs are divided into two categories, designated Class I and Class II, based on their amino acid sequence homology and structural topology [30, 40]. HA synthases belonging to Class I are present in some species of *Streptococcus*, viruses and vertebrates. Whereas, Class II HAS had so far only been described in *Pasteurella multocida* [30]. The latter differs from Class I HAS in terms of protein conformation and its relationship with the coupling of the plasma membrane, also affecting the mechanism of action of HA synthesis. Class I enzymes contain multiple transmembrane domains, while Class II HAS is coupled to the plasma membrane through a single domain near the carboxyl terminus by an undescribed mechanism [30]. Another main difference between the two classes of enzymes is the form of HA production, the enzymes of Class I add the two sugar precursors (UDP-GlcUA and UDP-GlcNAc) at the reducing end and the enzymes of the Class II extend the polysaccharide chain at the non-reducing end. Table 1 summarizes the main characteristics of HA synthases from Classes I and II.

**Table 1:** Main characteristic of HA synthases from Class I and II

	Class I		Class II
	Reducing	Nonreducing	
Organisms	*Streptococcus pyogenes*, *S*. *equisimilis*, *S*. *uberis*, mammalian and avian	Amphibian species, algal virus	HAS of *Pasteurella multocida*
Size (amino acids)	417–588		972
Membrane attachment domain	6–8 membrane-associated domains		*C*-terminal membrane anchor
HA chain growth	At reducing end	At nonreducing end	At nonreducing end
Reaction type	Hexosyl group transfer		hexosyl group transfer
Metals and ions	Co^2+^; KCl; Mg^2+^; Mn^2+^; NaCl		Co^2+^; Mg^2+^; Mn^2+^;
K_M_ value [mM]	0.032–1.1		0.014-0.91
Ki value [mM]	1.2–4.5		ND
pH optimum	5–9		ND
Temperature optimum (ºC)	22–60		ND

*ND* not determined

**Fig. 4:**
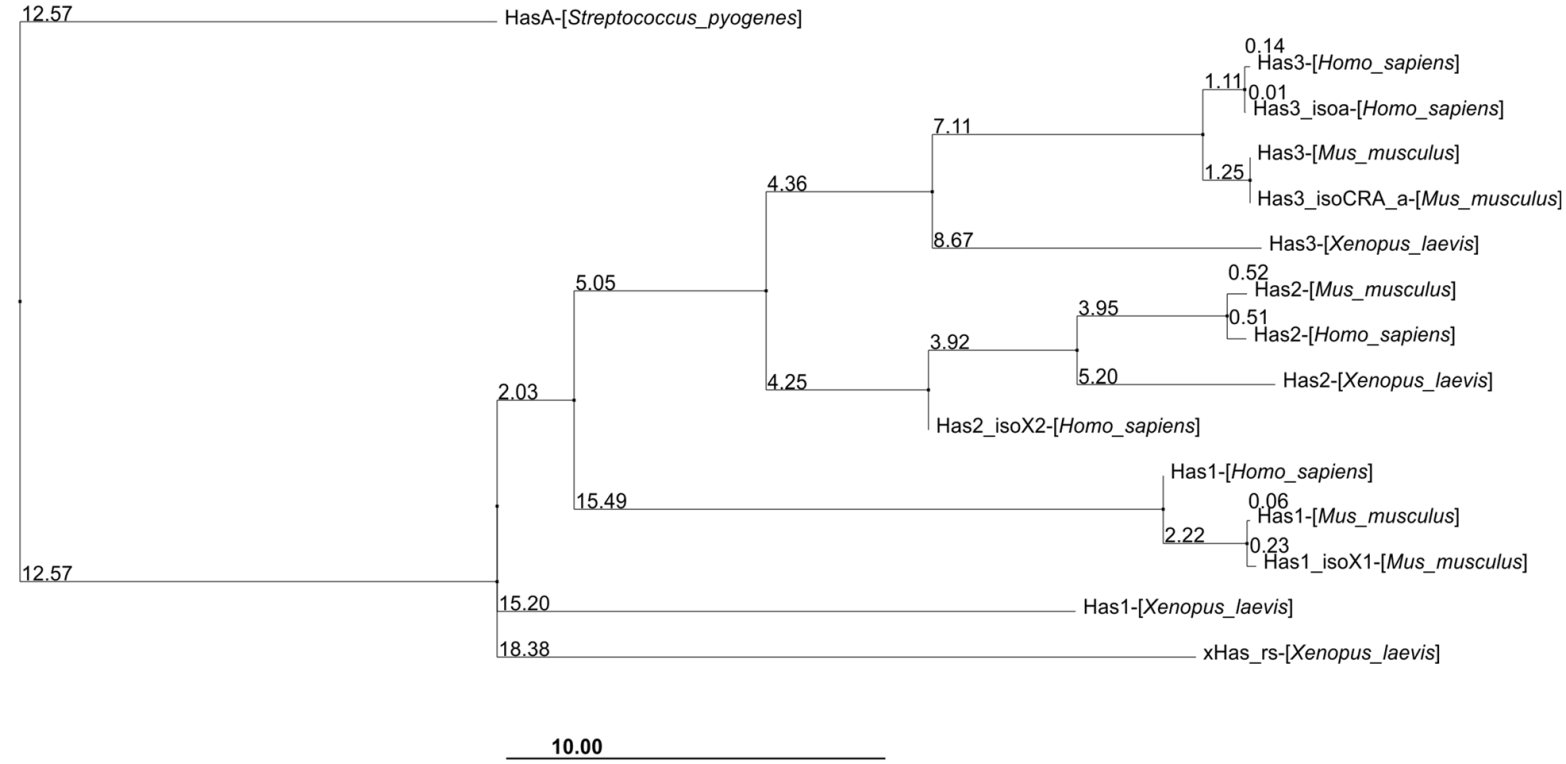
Evolutionary relationship between HA synthase proteins (HAS) in an identity percentage tree using Jalview 2.9.0b2. Acessions acquired through the NCBI BLAST tool, aiming for better score values in organisms, *Homo sapiens*, *Mus musculus*, *Xenopus laevis* aligned to the *Streptococcus pyogenis* protein sequence

The eukaryotic Class I HA synthases show homology to Class I HA synthases from microorganisms, indicated by up to five conserved transmembrane domains, with a DXD motif in the cytoplasmic region of the glycosyltransferase domain (PFam: PF00535) between the second and third transmembrane, therefore responsible for the binding of UTP-sugars to the enzyme leading to the polymerization of HA. The bacterial HAS demonstrates up to 47 % similarity within 92 % of query cover; this homology is usually attributed to a lateral gene-exchange from the animal host to the bacterium that may have occurred in the past. Figure 4 shows the phylogenetic relationship between streptococcal HAS protein sequences with the most relevant vertebrate model organisms in which HAS proteins have been reported. Hyaluronic acid produced by animals and microbes are extremely different in molecular weight and rate of synthesis, wherein the speed of synthesis in microbes is ten-fold faster than the speed in animals [25, 41]. However, the great similarity between the genes involved in the production of hyaluronic acid of different cells requires a theory for the existence of this sharing. The discovery that *Chrlorella* virus cells are capable of inducing the production of hyaluronic acid in host cells [42] and the discovery of a *has* gene into a *Bacillus anthracis* plasmid confirmed the theory and classification of the gene *has* as one of the 223 candidates that are capable of lateral gene transfer in bacteria to vertebrates [26], which explains the similarity existing among *has* genes. On the other hand, the Class II—HAS from *Pasteurella multocida* (pmHAS)—is different from all other HA synthases, as previously discussed, which is likely to be an example of convergent evolution, a phenomenon in which living organisms develop similar characteristics from different sources, in this case, different enzymes with the same product: HA [43].

It is now acknowledged that eukaryotes feature three HAS isoforms: HAS1, HAS2, and HAS3, encoded by three related *HAS* genes on three different chromosomes [2, 40]. The single exception is the frog, *Xenopus laevis*, for which four HAS encoding genes can be observed. Additionally, *X. laevis* HA synthase was reported to synthesize HA polymers of 20 MDa in vitro [2, 44], in contrast with the Streptococcal strains that generally produce smaller MW ranges of HA (around 0.8–1.5 MDa) and 3 MDa in animal tissues [45].

All the three isoforms of HAS in eukaryotes are owned to Class I and are structurally similar in relation to domains e regions. The three isoforms possess an *N*-terminal region, a cytoplasmic central region and a *C*-terminal hydrophobic region. The central region is the most well preserved and has a high rate of similarity between the three isoforms, reaching 87 %. However, for each reported isoform of HAS in eukaryotes, the catalytic rate and mode of regulation has been shown to be different [2, 46]. Although each of the three isoforms have a highly conserved amino acid sequences, each enzyme has different kinetic properties and mechanisms of action. HAS1 is an enzyme that has a lower activity and is responsible for the maintenance of constitutive levels of HA synthesis having a MW between 0.2 and 2 MDa; HAS2 is more active compared to HAS1, and generates HA polymers with MW greater than 2 MDa. In addition, HAS2 plays an important role in damage repair suffered by the tissues when they are in development and expansion, moreover, this isoform is involved in cardiac cushion morphogenesis. The enzyme HAS2 is also associated with the production of HA involved in cell migration and invasion, cell proliferation, and with angiogenesis during development. Being the more active isoform, HAS3 is able to produce HA chains of various sizes (0.2–2 MDa). The longer chains participate in the formation of the pericellular glycocalyx and are responsible for interactions with the cell surface receptors, while the shorter chains participate in processes that shape the cellular activities through signal transduction cascades.

Aiming to characterize and compare the enzymatic properties of the three HAS proteins, the three responsible genes for HA synthesis in mammals have been cloned and expressed either in COS-1 cell strain or rat 3Y1 fibroblast cell strain. Kinetic studies of these enzymes have shown distinctions between their stability, HA elongation rate and apparent K_M_ values for the UDP-GlcNAc and UDP-GlcUA substrates. When the study compared the V_max_ of the three recombinant isoforms (HAS1, HAS2, and HAS3), the values obtained were not significantly different (ranging from 330.5 to 398.5 pmol/h/unit) considering the concentrations of UDP-GlcUA as saturating (1.0 mM), whereas the Km value for UDP-GlcNAc of HAS1 was 1.01 mM; about three- to fourfold greater than those of HAS2 and HAS3, respectively [47]. This result strengthens the suggestion that this enzyme could be responsible for constitutive levels of HA as discussed earlier.

### Microorganisms that naturally produce HA

Some bacterial pathogens such as *Pasteurella multocida* and Gram-positive *Streptococcus* Group A and C can produce and secrete HA chains [48]. The particularities of each producing pathway from natural HA producers are detailed below. It is worth noting that some eukaryotic microorganisms also have the ability to synthesize HA, such as the yeast *Cryptococcus neoformans* and the green algae Chlorella sp., which occurs when it is infected by the *Paramecium bursaria* chlorella virus (or Chlorovirus) (PBCV-1) [49, 50].

HA synthesis from *Cryptococcus neoformans* is catalyzed by a glycosyltransferase (CPS1 gene) [49, 50]. Despite *C. Neoformans’* ability to synthesize HA, there are no reports involving *C. neoformans* as a large scale producer of this biopolymer, possibly for being an opportunistic pathogen [50, 51].

HA synthesis by microalgal Chlorella cells occurs upon infection with chloroviruses [48, 52]. PBCV-1-infected chlorella was monitored for HA synthesis, and studies indicated that about 80 % produce HA during infection [53]. In general, the production (maximum of 1 g/L, JP patent 2004-283096) does not reach the values typically achieved using Streptococcal fermentation (Table 2). However, recent methods were proposed to improve the yield of HA production in Chlorella: (1) using isolated chlorovirus cells that have low growth rate (CV01 and CVTS1) and (2) affecting the ability of cells to reproduce by using molecules capable of inhibit DNA synthesis inside the cell, and aphidicolin [52]. The use of the two approaches together allowed for a concentration of 14 µg/ml, about a 2.2-fold increase over PBCV-1 infected chlorella, which showed a concentration of 6.3 µg/mL HA.

**Table 2:** Organisms that naturally produce HA

Microorganism	Substrate concentration (g/L)	Production (g/L)	Yield Y_P/S_ (g/g)	Productivity (g/L/h)	Molecular weight (MDa)	Reference
*Streptococcus zooepidemicus*	Glucose: 10	0.95	0.09	0.0396	NR	[63]
*Streptococcus zooepidemicus* WSH-24	Glucose: 15	6.6	0.071	0.33	NR	[99]
*Streptococcus zooepidemicus* ATCC 39920	Glucose: 10	2.45	0.12	0.223	NR	[3]
*Streptococcus equi subsp. zooepidemicus* (*S. zooepidemicus* ATCC 35246)	Glucose: 20	NR	0.088	NR	2.4	[100]
*Streptococcus equi* ATCC 6580 (mutant)	Glucose: 80	5.5	NR	0.344	3.8	[57]
*Streptococcus equi* ATCC 6580 (mutant)	Glucose: 80	6–7	0.088^a^	0.47^b^	3.2	[57]
*Streptococcus zooepidemicus* G2 (mutant)	Glucose: 40	3.51	NR	0.251	2.19	[60]
*Streptococcus zooepidemicus* (overexpression of genes involved in biosynthesis of UDP-*N*-acetylglucosamine)	Glucose: 20	NR	0.075	NR	~3.4	[3]
*Streptococcus* sp. ID9102	Glucose: 40	6.94	NR	0.289	5.9	[101]
Chlorella cells infected with Chlorovirus	NR	0.5–1	NR	NR	NR	[27]
Chlorovirus infected with PBCV-1	NR	6.3 × 10^−6^	NR	1.6 × 10^−6^	NR	[41]
Chlorovirus with PBCV-1 CV01	NR	7.6 × 10^−6^	NR	0.95 × 10^−6^	NR	
Chlorovirus with PBCV-1 CVTS1	NR	9.2 × 10^−6^	NR	1.15 × 10^−6^	NR	
Chlorovirus infected with PBCV-1 (aphidicolin)	NR	~1.0 × 10^−5^	NR	5.3 × 10^−6^	NR	
Chlorovirus with PBCV-1 CV01 (Aphidicolin)	NR	14 × 10^−5^	NR	3.5 × 10^−6^	NR	
*Streptococcus* sp. ID9102 (KCTC 11935BP)	NR	6.94	NR	0.289	5.9	[101]

*NR* not reported

^a^Calculated from the values reported by the authors of the maximum production of HA (P) and the consumed substrate (S), defined as Yield = P/S

^b^Calculated from the values reported by the authors of the maximum production of HA (P) and the cultivation time to obtain this production (t), defined as productivity = P/t

### Pasteurella multocida

*Pasteurella multocida,* a non-motile, Gram-negative coccobacillus, is a pathogen that causes pneumonia in piglets and calves [54]. Synthesis of HA in *P. multocida* is catalyzed by a polypeptide originating from a single gene (pmHAS) [30, 43]. This gene encodes for a HAS enzyme that polymerizes the HA chain, adding the sugars glucose-6-phosphate and fructose-6-phosphate to the non-reducing end of the growing polymer chain. This characteristic allows the enzyme pmHAS to elongate exogenous and short chains of HA to form long chains in vitro. However, in vivo, the chains cannot be elongated due to a blockage of the enzyme by the actual HA chain [30].

In order to investigate which gene was responsible for HA synthesis in this microorganism, the entire capsule locus of *P. multocida* serogroup A:1 was cloned in *E. coli* DH5α and sequenced [55]. From this study, it was shown that only pmHAS was functional. However, sequence homology has identified hyaA and hyaC as a putative glycosyltransferase and a UDP-glucose dehydrogenase, respectively [55, 56]. Despite the fact that HA is naturally produced by *P. multocida*, there are no reports employing this microorganism to super-produce HA, possibly due to the microorganism’s pathogenicity or lack of genetic engineering tools. Nevertheless, it is more common to heterologously express pmHas genes for HA production in other microorganisms [57–59].

### Streptococcus *sp*.

*Streptococci* are non-sporulating and non-motile Gram-positive bacteria that have the characteristic to grow surrounded by a large extracellular capsule [20]. Various wild-type strains of Streptococci are able to produce HA, such as *Streptococcus equisimilis* (an animal pathogen) [60], *S. pyogenes* (a human pathogen) [31] and *S. uberis* (a pathogen in cattle) [61]. However, among these, the species *Streptococcus equi* subsp. *equi* and *S. equi* subsp *zooepidemicus* are the commonly used (Table 2).

The bacteria *Streptococcus zooepidemicus* (subspecie of *Streptococcus equi*) has a operon used for HA synthesis encoded by five genes: (1) HA synthase or HasA, (2) UDP-glucose dehydrogenase or HasB, (3) UDP-glucose pyrophosphorylase or HasC, (4) UDP-*N*-acetylglucosamine pyrophosphorylase or glmU that have a activity of acetyltransferase and pyrophosphorylase, and a gene encoding for (5) phosphoglucoisomerase [62, 63]. In *S. pyogenes*, the enzymes of the biosynthetic pathway of HA are regulated by a polycistronic mRNA, in other words, a single mRNA is transcribed from more than one gene, that includes HasA, HasB and HasC (involved in UDP-glucuronic acid synthesis) [64, 65] HasB is required to make UDP-GlcUA, one of the two substrates needed for HA synthesis, which is synthesized from glucose-6-phosphate coming of a deviation from the glycolytic route (via glucose-1-phosphate). HasC is an enzyme that catalyzes the conversion of UTP-Glc-1-phosphate (glycose-1-P) to UDP-Glucose (UDP-Glc), one of the two HA precursors (Fig. 3) [66]. HasA adds in strict alternating fashion the two HA precursors (UDP-GlcNAc and UDP-GlcUA) to the non-reducing end of the growing HA chain, using the two UDP-sugar substrates, alternatively creating β1 → 3 and β1 → 4 glycosydic bonds (Fig. 1) [2].

In addition to HA synthesis, many of these intermediate molecules are used by the cells as wall components (production of peptidoglycan) and teichoic acid components [25, 56]. Cloning of the streptococcal HA synthase encoding genes in *Escherichia coli* confirms that only HasA enzyme is required for HA biosynthesis when the cell is already producing glucuronic acid and *N*-acetylglucosamine [60].

Over time, a combination of strain improvement and cultivation conditions have been performed in order to enhance the HA production [67]. The mutant strain from *S. equi* ATCC 6580 was generated using sequential cultivations with *N*-methyl-*N*′-nitro-*N*-nitroso-guanidine and reached 6–7 g/L of HA in a 100 L fermentor (Table 2). In another study [63] a strain of *S. zooepidemicus* had the metabolic pathway of synthesis of HA engineered completely by an adjustment of the concentrations of the metabolites through the overexpression of the five HAS genes encoding for HA. The concentrations of the two precursors of HA within the cell affects the molecular weight of the final chains, when the genes involved in UDP-*N*-acetylglucosamine pathway are overexpressed the final HA MW increases (~3.4 MDa; double the amount found in wild-type strain), and when the genes involved in UDP-glucuronic pathway are overexpressed the final MW decreases (Table 2). On the other hand, overexpression of the enzyme that synthetizes HA (HasA) produces an increase in the HA yield [68].

The application of chemical mutagenesis followed by a serial selection program has been successfully exploited to obtain a hyaluronidase-negative, non-hemolytic, kanamycin-resistant, and highly viscous mutants strains of *S. equi* strains [67]. HA production by the *S. equi* ATCC 6580 mutant (renamed *S. equi* ATCC KFCC 10830) was tested with and without the addition of lysozyme. When lysozyme is added to the cells, the cell wall structure is disintegrated causing a stress to the culture broth during the growth, in these cases the HA production expanded of 3.65–4.63 g/L. This suggests that the HA is a cell protective agent produced by the microorganism in response to a unfavorable or critic environment [67] (Table 2).

*S. zooepidemicus* showed potential of HA production levels close to those obtained by means of processes that use high-value compounds used in the laboratory, while using marine industrial by-products as source of carbon and aminoacids [69]. The amount of dissolved oxygen in the culture medium also affects the production of HA, *S. zooepidemicus ATCC 39920* was exposed to ultraviolet (UV) light and *N*-methyl-*N*′-nitro-*N*-nitrosoguanidinea resulting in the mutant strain called G1 [70]. Under anaerobic conditions, cell growth and HA synthesis were suppressed and the HA MW was only 1.22 MDa with a final production of 0.73 g/L. On the other hand, the production of HA in an aerobic fermentation was obtained with a doubled MW, but there were no changes in biomass or HA yield (Table 2).

### Microorganisms genetically modified for HA production

Metabolic engineering has been providing opportunities to obtain HA from non-pathogenic, safe microorganisms and, hence, an endotoxin-free product since the natural producing organisms are mostly pathogenic. With these criteria, HA has already been produced by a wide range of heterologous hosts, including: *Lactococcus lactis* [71–75], *Enterococcus faecalis* [64], *Corynebacterium glutamicum* [76], *Agrobacterium* sp [58], *Escherichia coli* [43, 48, 64, 77–79]*, Streptomyces albulus* [80], *Bacillus subtilis* [74, 81, 82], *Saccharomyces cerevisiae* [83], *Pichia pastoris* [45] and plant cell cultures [84, 85]. Many studies have included the evaluation of HA production using different genetic modifications, HAS proteins, and different hosts as discussed below.

### Prokaryotic organism

Among the different domains, bacteria is the main exploited for heterologous production of HA. The following topics discuss some of the organisms studied so far.

#### Bacteria

##### *Lactococcus lactis*

Gram-positive lactic acid bacterium, *L. lactis* is used worldwide as starter for the production of numerous products [86]. *L. lactis* has many desirable characteristics for a fermentative microorganism and a GRAS status, therefore many attempts has been made to genetically modify it for HA production [72].

Nevertheless, *L. lactis* does not produce the enzyme HA synthase, which has a crucial role in HA production. Therefore, initial studies on HA production using *L. lactis* utilized non-integrative plasmids such as pRKN; pNZ8148; and their derivatives, for the expression of genes encoding HAS proteins [71–73, 75]. It is noteworthy that the *L. lactis* NZ9000 strain transformed with pSJR5, co-expressing five different genes (*hasA*, *hasB*, *hasC*, *hasD* and *hasE*) has exhibited structural and segregational instability, resulting in incorrect gene expression or loss of plasmid after few generations [71]. Strains successfully transformed with plasmids packed with genes *hasA*-*B*, *hasA*-*B*-*C*, *and hasA*-*B*-*D,* were respectively named strains SJR2, SJR3, and SJR6. Static flask experiments using glucose as a substrate indicated that the strain co-expressing the genes *hasA* and *hasB* obtained a production of 0.097 g/L HA, while the other experiment with the strain co-expressing *hasA*, *hasB* and *hasC* genes yielded a maximum production of 0.234 g/L HA, as indicated in Table 3, suggesting that the introduction of the *hasC* gene is essential for high HA production [71]. In submerged anaerobic fermentations, HA production was three times higher (0.72 g/L) using the SJR3 strain, in comparison to the SJR2 strain (0.26 g/L). Additionally, when the bioreactor containing a SJR3 culture was aerated at 1 vvm, HA production increased to 1.8 g/L. Another construct, named SJR6, was assessed for HA production and transcription of HA pathway genes, as shown in Table 3 [73].

**Table 3:** Prokaryotic microorganisms and their genetic modifications for the HA production

Microorganism	Genetic modification	Substrate concentration (g/L)	Production (g/L)	Yield Y_P/S_ (g/g)	Productivity (g/L/h)	Molecular weight (MDa)	Reference
*Lactococcus lactis* BCRC 12312	Operon containing HA synthase (*HasA*) of *S. equi* subsp. *zooepidemicus*	Glucose: 0.1	0.08	0.8^a^	0.0022^b^	NR	[62]
*Lactococcus lactis* BCRC 12312	Operon containing HA synthase (*HasA*) and UDP-GlcDH (*HasB*) of *S. equi* subsp. *zooepidemicus*	Glucose: 0.1	0.65	6.5^a^	0.018^b^	NR	[62]
*Lactococcus lactis* NZ9000	Expression of HA synthase and UDP-glucose-6-dehydrogenase of *Streptococcus zooepidemicus*	Fed batch of glucose: 5 and fed batch of lactose: 20	0.12	NR	NR	0.879	[65]
			0.59	NR	NR	0.569	
*Lactococcus lactis* NZ9000 (SJR2)	has operon of *Streptococcus zooepidemicus* (co-expressing HasA and HasB genes only)	Glucose: 15	0.107 (in static flask experiments)	0.007^a^	0.0178	NR	[61]
		Glucose: 25	0.068 (in static flask experiments)	0.002^a^	0.0113	NR	
*Lactococcus lactis* NZ9000 (SJR3)	has operon of *Streptococcus zooepidemicus* (co-expressing *HasA*, *HasB*, and *HasC* genes)	Glucose: 10	0.234 (in static flask experiments)	0.023^a^	0.039	NR	
		Glucose: 25	0.154 (in static flask experiments)	0.006^a^	0.257	NR	
*Lactococcus lactis* NZ9000 (SJR2)	has operon of *Streptococcus zooepidemicus* (co-expressing *HasA* and *HasB* genes only)	Glucose: 10	0.123	0.012	0.005^b^	NR	[63]
*Lactococcus lactis* NZ9000 (SJR3)	has operon of *Streptococcus zooepidemicus* (co-expressing *HasA*, *HasB*, and *HasC* genes)	Glucose: 10	0.43	0.041 (Y_P/S_)	0.0179^b^	NR	[63]
*Lactococcus lactis* NZ9000 (SJR6)	Three has operon genes (*HasA*, *HasB* and *glmU*) from *S. zooepidemicus*	Glucose: 10	0.595	0.09 g/g (Y_P/S_)	0.0248^b^	NR	
*L. lactis* NZ9000	L. lactis carrying *HasA*- *HasB* gene in *nisRK* region	Glucose: 10	0.14	0.014^a^	0.0117	4.3	[77]
VRJ2AB							
*Lactococcus lactis* NZ9000 VRJ3ABC	*L. lactis* carrying *HasA*- *HasB*- *HasC* gene in the *nisRK* region	Glucose: 10	0.68	0.068	0.0567	3.49	[77]
*Enterococcus faecalis* OGlRF	Transposon 916 insertional mutagenesis	THY broth	0.002-0.69	ND	ND	ND	[64]
	Was subcloned into a plasmid shuttle vector (pAT19 and pPD41)						
*Corynebacterium glutamicum* ATCC 13032 pJH174.1	*HasA* expression	MEK700 with glucose: 40	Between 0.3 and 0.4	0.017	~0.003^b^	>1.4	[87]
		CGXII with glucose: 40	~1.0	0.023	~0,008^b^	<0.27	
*Corynebacterium glutamicum* ATCC 13032 pJH181.3	*HasA* and *HasC* coexpression	MEK700 with glucose: 40	Between 0.3 and 0.4	0.016	~0.003^b^	>1.4	
		CGXII with glucose: 40	Between 1.0 and 1.3	0.025	~0.01^b^	<0.27	
*Corynebacterium glutamicum* ATCC 13032 pJH182.1	*HasA* and *vgb* (bacterial haemoglobin from *Vitreoscilla* sp.) coexpression	MEK700 with glucose: 40	Between 0.1 and 0.2	0.075	~0.001^b^	>1.4	
		CGXII with glucose: 40	~0.8	0.014	~0.007^b^	<0.27	
*Corynebacterium glutamicum* ATCC 13032 pJH183.2	*HasA*, *HasC* and *HasB* coexpression	MEK700 with glucose: 40	Between 0.3 and 0.4	0.017	~0.003^b^	>0.67	
		CGXII with glucose: 40	~1.3	0.027	~0.01^b^	<0.27	
*Corynebacterium glutamicum* ATCC 13032 pJH195.2	*HasA* and *glmU* (from *Pseudomonas* *putida* KT2440) coexpression	MEK700 with glucose: 40	~0.3	0.014	~0.003^b^	>1.4	
		CGXII with glucose: 40	Between 1.0 and 1.3	0.026	~0.01^b^	<0.27	
*Corynebacterium glutamicum* ATCC 13032 pJH196.2	*HasA*, *HasC* and *glmU* coexpression	MEK700 with glucose: 40	Between 0.3 and 0.4	0.017	~0.003^b^	>1.4	
		CGXII with glucose: 40	~1.2	0.027	~0.01^b^	<0.27	
*Corynebacterium glutamicum* ATCC 13032 pJH197.1	*HasA*, *glmU* (from *Pseudomonas putida* KT2440) and *HasC* coexpression	MEK700 with glucose: 40	<0.3	0.013	~0.003^b^	>0.67	
		CGXII with glucose: 40	Between 1.0 and 1.3	0.025	~ ~0.01^b^	<0.27	
*Agrobacterium* sp ATCC31749	Coexpression of HA synthase from *Pasteurella multocida*, and UDP-glucose dehydrogenase from *Escherichia coli*	Sucrose: ~42.8, Lactose: 3.4	~3.0	0.0645^a^	0.5^b^	1.56	[47]
*Agrobacterium* sp LTU261	Coexpression of HA synthase from *Pasteurella multocida*, and UDP-glucose dehydrogenase from *Escherichia coli*		~2.3	0.0495^a^	0.38^b^	2.17	
*Agrobacterium* sp LTU265	Coexpression of HA synthase from *Pasteurella multocida*, and UDP-glucose dehydrogenase from *Escherichia coli*		~2.4	0.06^a^	0.4^b^	0.72	
*Escherichia* *coli* JM109	Co-expression of HA synthase from *Pasteurella*	Terrific Broth/feeding medium contained 0.50 glucose (after feeding: glucose: 50) (Bioreactor)	2.0	0.027	0.0127	NR	[48]
			3.8 (fed-batch fermentation process in a 1 L				
	*Multocida* and uridine diphosphate-glucose dehydrogenase from *E. coli* K5 strain		Bioreactor)				
*Escherichia coli* OP50	CPS1 cDNA from *C. neoformans*	NR	NR	NR	~4 ng/hr/µg protein	NR	[39]
*Escherichia coli* x1448	Transposon 916 insertional mutagenesis was subcloned into a plasmid shuttle vector (pPD41, pPD41Δ4, pPD41 Δ5 and pPD41 Δ6)	THY broth	0.002–0.08	NR	NR	NR	[64]
*Escherichia* *coli* K12 MG1655 sseAB	Gene of sse HasA with identical protein sequence of seHAS from *Streptococcus equisimilis*	Glucose: 10 (fed batch)	0.155 (24 h)	0.0155 (24 h)^a^	0.0065 (24 h)	0.38 (24 h)	[69]
			0.196 (48 h)	0.0196 (48 h)^a^	0.0041 (48 h)	0.5 M (48 h)	
			0.202 (72 h)	0.0202 (72 h)^a^	0.0028 (72 h)	1.7 (72 h)	
*Escherichia coli* K12 MG1655 sseABC	Gene of sse HasA with identical protein sequence of seHAS from *Streptococcus equisimilis*	Glucose: 10 (fed batch)	0.148 (48 h)	0.0148^a^	0.0031	0.39	[69]
*Escherichia* *coli* C0 Top10/(pMBAD-sseABC, pHACM-blank)	Two plasmids and the chromosomal copies of wildtype rpoD and rpoS.	Glucose: 10 (fed batch)	0.405	0.0405^a^	0.008	NR	[92]
*Escherichia* *coli* C1 Top10/(pMBAD-sseABC)	Used as the control strain in library screening, harboring a single plasmid and the chromosomal copies of wild type rpoD and rpoS	Glucose: 10 (fed batch)	0.510	0.0510^a^	0.0106	NR	[92]
*Escherichia* *coli* D72 Top10/(pMBAD-sseABC, pHACM-rpoDM72)	Extra copy of mutant rpoD or rpoS in the pHACM plasmid	Glucose: 10 (fed batch)	0.561	0.0561^a^	0.0117	NR	[92]
*Escherichia* *coli* D2 Top10/(pMBAD-sseABC, pHACM-rpoDM2)	Extra copy of mutant rpoD or rpoS in the pHACM plasmid	Glucose: 10 (fed batch)	0.548	0.0548^a^	0.0114	NR	[92]
*Escherichia* *coli* S47 Top10/(pMBAD-sseABC, pHACM-rpoSM47)	Extra copy of mutant rpoD or rpoS in the pHACM plasmid	Glucose: 10 (fed batch)	0.479	0.0479^a^	0.00998	NR	[92]
*Escherichia* *coli* D0 Top10/(pMBAD-sseABC, pHACM-rpoD)	With an extra copy of unmutated rpoD on pHACM;	Glucose: 10 (fed batch)	0.425	0.0425^a^	0.009	NR	[92]
*Escherichia coli* S0 Top10/(pMBAD-sseABC, pHACM-rpoS)	With an extra copy of unmutated rpoS on pHACM;	Fed batch of glucose: 10	0.696	0.0696^a^	0.015	NR	[92]
*Streptomyces albulus* pJHA4	hasA gene from 29 *Streptococcus zooepidemicus*	Fed batch of glucose glucose: 50	6.2	0.062^a^	0.886^b^	2	[70]
*Bacillus subtilis*	Operon containing HA synthase (*HasA*) of *Streptococcus*, tuaD (*hasB*)	Fed batch rate of sucrose: 2	NR	1	NR	4.0	[71]
*Bacillus subtilis*	Operon containing HA synthase (*HasA*) of *Streptococcus*, tuaD (*hasB*) and Vitreoscilla hemoglobin (*VHb*)	Glucose: 10	1.8	0.18^a^	0.6^b^	NR	[64]
*Bacillus subtilis*	Operon containing HA synthase (*HasA*) of *Streptococcus* and tuaD controlled by inductive promoters	Glucose: 10	6.8	0.68^a^	0.34^b^	6.5	[72]

*NR* not reported

^a^Calculated from the values reported by the authors of the maximum production of HA (P) and the consumed substrate (S), defined as Yield = P/S

^b^Calculated from the values reported by the authors of the maximum production of HA (P) and the cultivation time to obtain this production (t), defined as Productivity = P/t

The low levels of HA production conducted with the SJR2 strain was hypothesized to be due to low UDP-glucose pyrophosphorylase (*hasC*) levels [71]. On the other hand, the co-expression of the genes *hasC,hasA* and *hasB* genes (SJR3 strain), increases HA production from 0.097 to 0.234 g/L. It has been discussed that increased HA production levels are due to the increase in UDP-glucose pyrophosphorylase levels in the SJR3 strain culture, diverting the flux of glucose-1-phosphate toward UDP-glucose synthesis. Therefore, different concentrations of substrate were tested for each recombinant strain, all listed in Table 3. From these results, it is assumed that HA biosynthesis depends not only on culture conditions and on the combination of heterologous genes incorporated in the strain but also on the expression level and transcriptional regulation of the homologous genes in the host genome.

The plasmids pEIrkA, pEIrkB, and pEIrkAB containing *hasA*, *hasB*, and *hasA* together with *hasB*, respectively, all from *S. equi* subsp. *Zooepidemicus*, have been introduced into *L. lactis* [72]. The expression of each gene was studied in order to relate it to HA production levels. The strain containing only *hasA* produced 0.08 g/L, while the strain containing *hasA* together with *hasB* had approximately an eight-fold increase in HA production, as shown in Table 3. The strain containing only *hasB* did not produce any HA [72].

Recombinant *L. lactis* strains were developed, resulting in two strains called VRJ2AB, carrying *HasA* and *HasB* genes, and VRJ3ABC, carrying *HasA*, *HasB* and *HasC* genes, integrated into their genomes [87]. HA production and MWs were, respectively, between 0.14 and 0.68 g/L and 4.3 and 3.49 MDa (Table 3). Overall, genome-integrated strains produced a two-fold increase in the HA polymer MW when compared to the plasmid-based strains. The significant difference in MW of HA derived from these strains could be explained by the precursors ratio effects (UDP-GlcNAc/UDP-GlcUA) and the *HasA*/*HasB* mRNA ratio. In the plasmid-bearing strains, *HasA* gene expression was high, but due to lower *HasB* expression, a sufficient substrate was not available during synthesis of HA chains by HA synthases, resulting in a relatively lower MW. In genome-integrated strains, there were relatively fewer HA synthases available and a greater availability of precursors for binding to HA synthase, facilitating greater MWs to be synthesized. In another study, it was hypothesized that the MW of HA can be regulated by *hasA*/*hasB* mRNA production levels. When the *hasA*/*hasB*-mRNA ratio was above 1.00, the HA polymer usually had a smaller size when compared to ratios below 1.00, whereby *hasB* mRNA levels are greater than *hasA* [75].

##### *Enterococcus faecalis*

This bacterium is a Gram-positive cocci with a natural habitat in the oral cavity and human intestinal lumen [88]. In the literature, there are only a few studies involving *E. faecalis* and the production of HA [89]. This might be due to the low total production yield obtained when compared to other microorganisms as shown in Tables 2 and 3. De Angelis et al. reported that the introduction of locus encoding of at least two streptococcal proteins could produce HA in acapsular *E. faecalis* mutants [89]. When HasA was introduced in *E. faecalis*, it was able to synthesize 0.69 g/L HA using 20 g/L of glucose (Table 3).

##### *Corynebacterium glutamicum*

These Gram-positive bacteria are the important microorganism with GRAS status for industrial amino acid production [90]. Nevertheless, *C. glutamicum* is also used for the biosynthesis of pantothenic acid [91], carotenoids [92], organic acids [93] and biofuels [94–96].

Therefore, for HA heterologous production, *C. glutamicum* was tested as an alternative host [76]. For that, a set of expression vectors was constructed containing HasA, encoding HA synthase from *S. equi* subsp. *Zooepidemicus*. To analyze the influence of precursors concentrations in the metabolic pathway, some vectors were constructed containing the genes *HasB*, *HasC*, and *glmU* (from *Pseudomonas putida* KT2440 strain) or hemoglobin from bacteria (vgb from *Vitreoscilla* sp.) [97]. The strains which co-expressed *HasB*, *HasC* or *glmU* had no result on HA yield and did not improve the MW of the product (Table 3). In contrast, co-expression of vgb decreased HA yield approximately 1.5-fold and did not affect the MW of the product. This study also analyzed how the media composition affect the HA production and MW, and observed that when using the medium CGXII, a production of 1.2 g/L was achieved, while the MEK700 medium production was almost 3.5 times less. On the other hand, the MW obtained in the MEK700 medium was greater (1.4 MDa) than was obtained in the CGXII medium (<0.27 MDa) (Table 3).

##### *Agrobacterium* sp.

The *Agrobacterium* ATCC31749 strain is known as a curdlan polysaccharide producer. The efficient production of this glucose polymer implicates an effective mechanism for sugar nucleotide UDP-glucose synthesis, demonstrating a natural tendency for synthesizing the sugar nucleotide precursor, UDP-glucose [98].

However, HA production studies using *Agrobacterium* as the host are limited. A single study explored three *Agrobacterium* strains as the host for HA synthesis through the expression of the *pmHas* gene from *P. multocida*: *Agrobacterium* sp. ATCC31749, an overproducing curdlan strain and *Agrobacterium* sp. LTU261 and LTU265, with defects in curdlan synthesis regulation and transport, respectively [58]. The recombinant strains had the ability to synthesize HA with production levels resulting in 3.0, 2.3 and 2.4 g/L, respectively, and with a MW around 1.56, 2.17 and 0.72 MDa, respectively, as indicated in Table 3. The HA MW produced from *Agrobacterium* sp. LTU261 was approximately 1.3 times greater than commercial HA extracted from *Streptococcus* (Table 3).

##### *Escherichia coli*

The first use of recombinant *E. coli* for HA production was to validate the function of the encoding gene for the HAS from *S. pyogenes* Group A [64]. In the early 2000s, the use of a recombinant *E. coli* showed the possibility of producing human HAS [40]. Therefore the first human HAS successfully expressed in *E. coli* was the catalytic region of human HAS2 isoform. Nevertheless, the levels of HA produced were not reported in this study [40]. In 2007, CPS1 cDNA from *C. neoformans* was expressed in *E. coli* OP50 and resulted in a strain that was able to synthesize HA with a productivity of 4 ng/hr/µg total protein (Table 3) [50].

In recent years, more efficient production in *E. coli* has been achieved through codon optimization, overexpression of HasB and random mutagenesis, as shown in Table 3 [79, 99]. Different fermentation profiles have shown that HA MW and concentration were increased from 0.38 to 1.9 MDa and from 0.148 to 0.202 g/L, respectively [79] (Table 3).

In a follow up study, a strain of *E. coli* was successfully engineered to produce HA through the expression of *pmHas* gene from *P. multocida* subsp*. multocida* [59]. The strain produced about 0.5 g/L HA cultured in shake flask and about 2.0–3.8 g/L in a fed-batch fermentation process in a 1-L fermentor. In the same year, HA production was increased in an *E. coli* strain, JM109, modified to co-produce two enzymes, *P. multocida* HA synthase and *E. coli* K5 UDP-glucose dehydrogenase.

##### *Streptomyces albulus*

This soil-dwelling actinomycete is known for producing a wide range of bioactive secondary metabolites [100]. It has been proposed that enhancing the amount of intracellular ATP is a good strategy to achieve higher MW HA and improve the productivity in microorganisms [80]. Considering this, *S. zooepidemicushasA* gene was modified and expressed in the *S. albulus* pJHA4 strain, which has the potential to generate ATP at high levels, under the regulator of a late-log growth phase-operating promoter. This resulted in efficient production of HA in the 2.0 MDa MW range, which is greater than typical bacterial HA (which ranges from 0.5 to 2.0 MDa), indicating that the increased amount of intracellular HA precursors, through higher levels of ATP available to the cell, can lead to increased HA production, achieving 6.2 g/L after 72 h fermentation.

##### *Bacillus subtilis*

This bacterium is a Gram-positive, spore-forming microorganism found in soil, water and in association with plants. It is one of the most broadly used models for genetic engineering. This bacteria has been utilized for the production of pharmaceuticals due to its well-characterized production of secondary metabolites that can be used as antimicrobial agents [101] and surfactants [102], besides being an important enzyme producer. The use of a *B. subtilis* strain is an excellent strategy for HA production since *B. subtilis* has GRAS status, ensuring that endotoxin-free products can be developed in industrial-scale.

A recombinant *B. subtilis* strain was developed, expressing *S. equisimilis* HA synthase gene, hasA, resulting in the production of HA with MW values around 1 MDa (Table 3). The association of hasA gene with other genes related to the biosynthesis of UDP-precursors has been tested, resulting in different operons, transformed in recombinant *B. subtilis* strains. It was observed that UDP-glucuronic acid level is a limiting factor for the HA production, in *B. subtilis.* The strategy used for the development of the recombinant strains was based on the identification of hasB homologous genes in *B. subtilis* (*hasB* and *tuaD*) and then overexpressed aiming at increased production level of intermediates for HA biosynthesis. After that, hasA was cloned under the control of a strong *S. equisimilis* promoter, called *amyQ*. The product obtained in the *B. subtilis* system was confirmed to be secreted and of similar quality, compared to commercial products [81].

In another study, a plasmid containing a hasA gene and the hasB gene from *Streptococcus* or tuaD (the same activity of hasB from *Streptococcus*) in *B. subtilis* were integrated into the amyE locus of the *B. subtilis* chromosome [74]. Within this construction, all genes were under the control of a strong constitutive promoter named VegII from *B. subtilis*. They coexpressed Vitreoscilla hemoglobin (VHb) in a *B. subtilis* concomitant to the HA-encoding genes which resulted in a 25 % increased growth rate as well as double the HA production. The strain expressing VHb, hasA and tuaD under the same regulation obtained HA in a concentration of 1.8 g/L after 30 h of cultivation (Table 3). It has also been shown that cells of *B. subtilis* containing the expression cassette with the tuaD gene (1.14 g/L) are 30 % more efficient in producing HA than cells containing only the *hasB* gene (0.84 g/L) [74].

More recently, a two-stage induction strategy has been utilized in the *B. subtilis* 168 (BGSC strain) aimed at increasing HA yield [82]. In this study, two constructions were used: (1) plasmid pAX01, which was used for cloning the HA synthase gene from *P. multocida* (PmHAS); (2) plasmid pHCMC05, which was used for the construction of recombinant operons for the enzymes related to the synthesis of the UDP-precursor sugars. The TPG223 strain was obtained after transformation with both vectors, pAX01-PmHAS and pHCMC05-tuaD-gtaB (tuaD and gtaB coding for UDP-GlcUA biosynthesis), this strain achieved HA production of 6.8 g/L and MW of 4.5 MDa (Table 3). Another strategy was designed to test for the UDP-GlcNAc, adding the gcaD gene to the system, gcaD coding for products related to UDP-GlcNAc biosynthesis. This strain, PG6181, obtained HA production of 2.4 g/L and MW of 0.013 MDa (Table 3). The results observed in those studies point to the relevance of UDP-GlcUA levels to the overproduction of HA in *B. subtilis* expression system.

#### Eukaryotic organisms

Numerous bacterial systems for the heterologous production of HA have been presented. Nevertheless, in the last few years, the use of eukaryotic organisms has increased. Here we introduce some eukaryotic systems described in the literature that are used for the production of HA.

##### Yeasts

Technologies that use yeast for the production of HA are more advantageous over other methods that are currently available on the market. For instance, there are yeast species that are not pathogenic, and its use for HA production would decrease downstream costs. Additionally, the extensive genomic knowledge of various yeasts together with available genetic tools allows target genetic modification for heterologous production of HA. Moreover, some yeast species already produce the intermediates for HA production such as glucuronic acid and *N*-acetylglucosamine and, in theory, in those species that require fewer genetic modifications. Finally, some yeast species are already widely used in many industrial processes with known cultivation technologies which could also reduce costs in large-scale HA production. HA production in yeast is not as characterized as in bacteria, however, there are some studies which indicate the possibility of using yeast for the heterologous production of HA.

##### *Saccharomyces cerevisiae*

This yeast is widely used in industrial processes. Its biology is the most understood of all the yeasts due to its utilization for beer, bread and wine production. Nevertheless, *S. cerevisiae* does not produce HA naturally. Therefore, it was genetically modified nearly 20 years ago for the production of HA [83]. For that, the hasA gene DG42 from *Xenopus* was introduced into the *S. cerevisiae* strain using pYES2, an epissomal plasmid. The recombinant *S. cerevisiae* INSc1 strain containing the plasmid has been shown to incorporate glucuronic acid and *N*-acetylglucosamine from exogenously supplied UDP-sugar nucleotides into a high MW polymer and has produced about 1–10 MDa [83].

##### *Pichia pastoris*

The development of recombinant *P. pastoris* for HA production has been recently described [45]. In this study, strains have been modified with the introduction of five genes: *HasC*, *glmU* (pyrophosphorilase), *pgI* (phosphoglucoisomerase), *HasA* and *HasB* (Fig. 3). In this study, two types of plasmid were utilized: the vector pAO815 and the vector pGAPZB, which have an inductive promoter (AOX) and a constitutive promoter (GAP), respectively. Cassettes containing *HasB*, *HasC*, *glmU* and *pgi* were inserted in different combinations into pGAPZ B, whereas cassettes containing *HasA*, *HasB*, *HasC*, *glmU* and *pgi* were inserted into different combinations in pAO815 and both vectors were inserted into *P. pastoris*. The idea was to use the vector containing constitutive promoters to cause an accumulation of both acid precursors in the cell and in a second step activate the inductive promoter preceding the *HasA* gene, leading to increased HA production and higher MW HA. The strategy also included performing fermentation of *P. pastoris* at a temperature below the optimal growth temperature to avoid a deviation of the carbon flux from the acid precursors for the wall synthesis and consequently increase the production yield of HA. This strategy achieved production values between 0.8–1.7 g/L of HA with a MW in the range of 1.2–2.5 MDa.

### Plant cell culture

Plant cell systems for the production of industrial materials show advantages over bacterial and mammalian vectors, such as: (1) the lack of human transmissible viruses and no risk of spongiform encephalopathies transmission; (2) costs related to the production are lower when compared to conventional fermentation processes; (3) as a photosynthetic system which fixates carbon dioxide, can be considered eco-friendly, reducing global warming gases levels [85].

Hyaluronidase is an enzyme that depolymerizes HA and thus increases membrane permeability, decreases viscosity, and makes tissues more readily permeable [103]. It can be used as a “spreading factor”, enabling the diffusion of molecules through the tissues [104]. Four genes coding for hyaluronidases in humans*, rHuHyal*-*1*, -*2*, -*3* and -*4*, have been successfully expressed and purified using *Nicotiana benthamiana* as host [84]. Experiments performed with those enzymes demonstrated that post-translational protein modifications patterns, biochemical properties and activities were similar to those shown by animal isolated hyaluronidases.

After the transformation of tobacco-cultured cells (BY-2) with a chloroviral HA synthase (cvHAS) gene to produce HA, HA was detected, but not measured by the authors [85].

### Patent publications

**Fig. 5:**
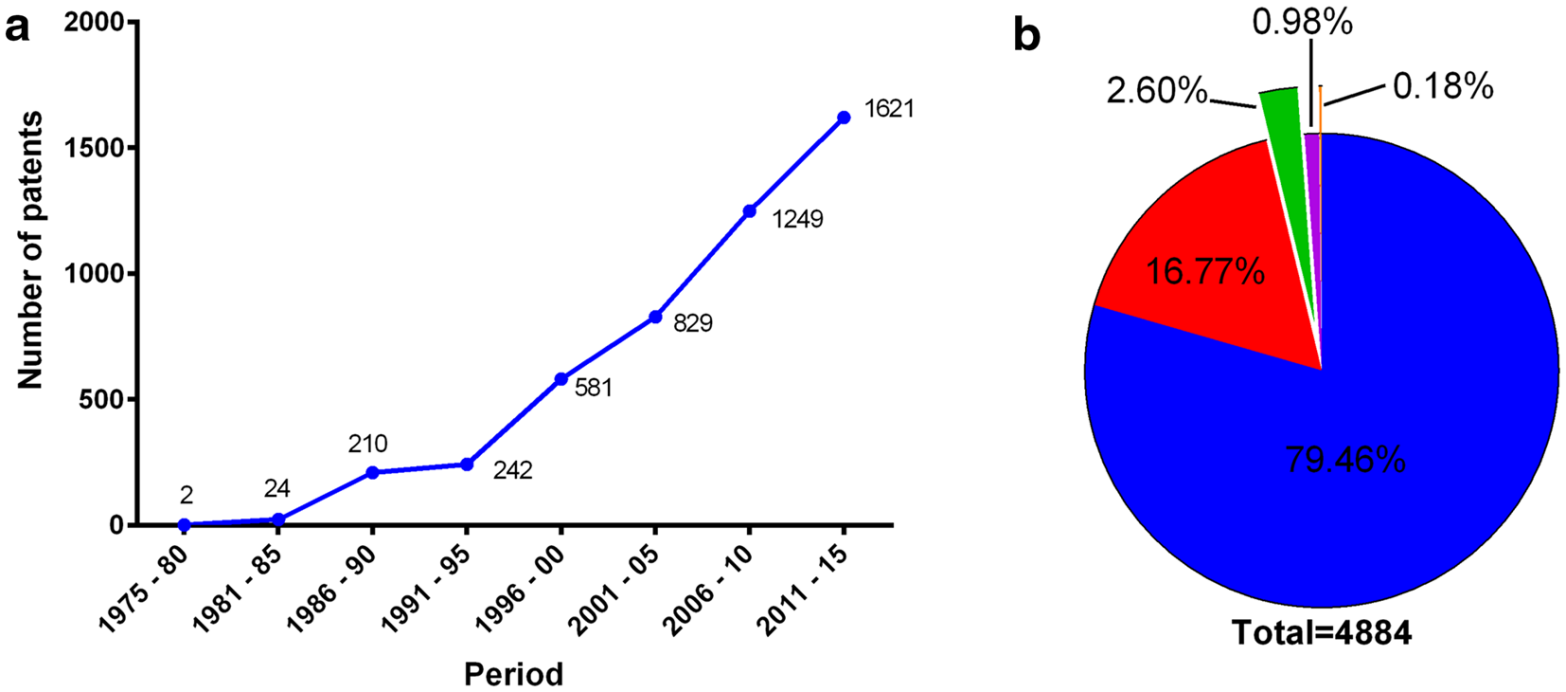
Patents: **a** Number of patent publications involving hyaluronic acid since 1975, in the world. **b** Key areas covered by the hyaluronic acid patent market. In *blue* are the patents involving preparations for medical purposes, in *red* are the patents involving processes for techniques used in Biochemistry, in *green* are the patents involving foods and foodstuffs, in orange are the patents involving processes for the development of hyaluronic acid chains and in *yellow* are others Source: Espacenet Patent Database. Accessed on 02/11/2015

In accordance with a database containing over 80 million patent documents from about 90 different countries, the number of patent documents containing “hyaluronic acid” in the title is growing every year, as shown in the Fig. 5a. There is a total of 4844 patents containing “hyaluronic acid” (or synonymous words) in the publication title. Most of these publications are related to the development of medical applications and formulations, followed by food and chemical industries (Fig. 5b). Among the techniques used in medical formulations, more than half are associated with the production of cosmetic preparations (62 %), followed by preparations for use in surgeries in various areas (28 %), such as ophthalmology. Still, in relation to patent registry for the production of HA, there is a severe disadvantage in price and purification of HA extraction via animal origin, thus, many methods using plants and microorganisms for the production of HA have emerged. According to a search in the same patent databases listed above, there are different projects using host cells for the production of HA, as illustrated in Table 4.

**Table 4:** Engineered organisms patented for hyaluronic acid synthesis

Host cell	Patent number	Patent date
*Escherichia coli*	US2014099673 (A1)	10/04/2014
	EP2614088 (A1)	17/07/2013
	CN102154190 (A)	17/08/2011
*Bacillus subtilis*	EP2614088 (A1)	17/07/2013
*Bacillus megaterium*	US2014099673 (A1)	10/04/2014
*Streptococcus zooepidemicus*	CN103993031 (A)	20/08/2014
*Streptococcus thermophilus*	JP2012130287 (A)	12/07/2012
*Pichia pastoris*	CN104212732 (A)	17/12/2014
Yeast cell^a^	JP2007174957 (A)	12/07/2007
Alga cell	EP2914716 (A1)	09/09/2015
Plant cell	PT1951878 (E)	08/06/2015
	AU2013201153 (A1)	21/03/2013
	US2009260108 (A1)	15/10/2009
	US2009199311 (A1)	06/08/2009

^a^Yeast specie not informed

## Conclusions and perspectives

HA has innumerous biological uses in the human body, from signaling processes during embryonic development to wound healing. Furthermore, it is important for the treatment of arthritis and osteoarthritis. Additionally, it has high commercial value compared to extracellular polysaccharides obtained from other microorganisms.

Previously, HA used to be extracted from animal waste. Nowadays, it is being replaced by production through bacterial fermentation. However, the fact that the microorganisms that naturally produce HA are pathogenic has stimulated studies to obtain this biopolymer using non-pathogenic and industrially friendly microorganisms. Nevertheless, up to now there has been no heterologous host producing as much HA as the natural ones. However, this fact has not discouraged researchers from attempting to obtain an ideal host for the production of heterologous HA. Rather, the search for this organism has included a wide variety of organisms such as bacteria, yeast, plants and virus-infected algae.

Besides the importance of heterologous production of hyaluronic acid by GRAS microorganisms, the research involving this polymer should advance to overcome the challenges regarding the metabolic route of its production. For example: (1) the competition between hyaluronic acid synthesis and cell growth (cell wall biosynthesis) observed in all the producers; (2) the inversely proportional relationship between the high concentration production and high MW; (3) the limitation of the fermentation in the bioreactor when it reaches 10 g/L due to the high viscosity of the medium; (4) the control of the amount of *has* gene transcribed, that directly affects the production of hyaluronic acid and the health of the cell wall (which can make unviable cells); (5) the control and equilibrium of the amount of the both precursors necessary for hyaluronic acid synthesis, where it is previously described that low concentrations of *N*-acetylglucosamine causes an inhibition of the synthesis, (6) the co-production of molecules like lactic acid that inhibits cell growth due to lower pH, and, lastly, (7) the balance between the recycling of cofactors (ATP, UTP and NADH) available in the cell for the use in the biosynthetic processes. All these challenges require specific studies to increase the knowledge for the HA optimal production conditions.

Furthermore, the advances in genetic engineering tool, especially in the genome editing area should contribute for the development of novel strains over producing HA. Recently, CRISPR/Cas9 technology is attracting more space withint the scientific community [105]. After the first use, CRISP-CAS9 has been used in a wide range of microorganisms, including the bacteria *Escherichia coli* [106] and yeast *Saccharomyces cerevisiae* [107].

However, in literature, there are no reports of any use of CRISPR/Cas9 technology involving heterologous production of hyaluronic acid, perhaps by the very recent nature of the technology. All hyaluronic acid studies involve traditional technologies, such electroporation techiques, homologous recombination in yeast [45] and gene insertion via plasmid [81]. The possible use of CRISPR/Cas9 in the production of hyaluronic acid could generate some advantages, including: (1) gene insertion in specific regions, such close of strong promoters, (2) considerable increase efficiency of clones with *has* gene, (3) regulation of genic transcription through the inclusion of transcription factors and (4) repression of genes that act directly or indirectly by inhibiting the synthesis of hyaluronic acid.

Among the microorganisms studied in this review, the yeast *P. pastoris* has been a host with commercial potential to produce HA. This is due to the combination of its production reaching an output of 0.8–1.7 g/L with a MW from 1.2 to 2.5 MDa [45]. Therefore, further studies on heterologous production using *P. pastoris* as a host may allow for greater production yields.

It is worth emphasizing that the ideal molecular size of HA will depend on its application. For example, in order to promote the healing of skin wounds and venous leg ulcers and to manage chronic wounds, a high MW is needed, while in rheumatoid and osteoarthritis, the MW and concentration of synovial fluid-HA are reduced. Additionally, the production mode can interfere directly in the production yield. Therefore, considering a culturing process using two stages (growth and production) could increase HA production, because there is a competition of HA precursors, which are also required for cell wall synthesis, for example.

It is noteworthy that the elucidation of biosynthetic pathways for HA-producing microorganisms and the use of genetic engineering combined with the optimization of biotechnological processes certainly corroborate with the increase of such outcomes in the heterologous production of HA. Therefore, the future of HA production process will certainly be the association of metabolic engineering and process design strategies.

## Abbreviations

HA: hyaluronic acid
UDP-GlcUA: uridine diphosphate glucuronic acid
UDP-GlcNAc: uridine diphosphate *N*-acetylglucosamine
HAS: hyaluronic acid synthases
GRAS: generally regarded as safe
MDa: megadalton
MW: molecular weight
ATP: adenosine triphosphate
UTP: uridine triphosphate
NAD^+^: nicotinamide-adenine dinucleotide
Acetyl-CoA: acetyl coenzyme A
pmHAS: *Pasteurella multocida* hyaluronic acid synthase
Km: Michaelis constant
V_max_: maximum reaction velocity
